# Imaging global auroras in space

**DOI:** 10.1038/s41377-019-0197-z

**Published:** 2019-09-11

**Authors:** A. T. Y. Lui

**Affiliations:** JHU/APL, Laurel, MD 20723-6099 USA

**Keywords:** Optics and photonics, Physics

## Abstract

China’s initial participation in the global monitoring of auroras for scientific and space weather investigations has been enabled by the successful launch of the Chinese Fengyun-3D satellite, which carries a wide-field auroral imager.

Auroral displays are one of the natural wonders of the world. Not only do these auroras display brilliant rainbow-like lights, but they also impress viewers with dynamic patterns, outshining the static display of a rainbow. At times, auroras appear as curtains stretching from horizon to horizon across the sky, as rays emanating from an invisible object, as spirals covering the entire sky, or as dynamic flickers and patches appearing chaotically in distributed areas, mimicking uncoordinated fireworks.

The space age began with the launch of the Russian artificial satellite Sputnik-1 on 4 October 1957, followed by the launch of the American artificial satellite Explorer 1 on 1 February 1958. Space scientists then recognized the prospect of direct measurements in space. Along with these exciting developments, the International Geophysical Year (IGY) was established, promoting worldwide scientific collaborations. Sixty-seven countries participated in this interchange. Auroras were one of the 11 Earth science topics encompassed by the IGY projects. It soon became evident that this international endeavor could best be accomplished by coordinated networks of all-sky cameras to monitor auroral activity over the entire polar region. Subsequent scientific progress has led to the realization that global auroral activity involves a two-dimensional projection of electromagnetic disturbances over the vast three-dimensional volume of space surrounding the earth.

Technological advances in the modern era have brought to our society numerous space developments that play a significant role in our daily life, in fields such as communication, banking, navigation, and global atmospheric weather surveillance.

Disadvantages arise for global observations of auroras from the ground. These observations are hampered by adverse weather conditions such as snow, haze, and clouds, as well as by moonlight. Satellite imaging of auroras can avoid these obstacles. The first scientific satellite to carry an auroral imager was the Canadian International Satellite for Ionospheric Studies (ISIS) 2, which was launched on 1 April 1971, in a nearly polar circular orbit with an inclination of ~88° at an altitude of ~1400 km and an orbital period of ~114 min. The auroral scanning imager possesses two opposite viewing apertures, one fitted with a 557.7-nm filter and one with a 391.4-nm filter^1^. The combined internal electronic scanning, satellite spinning, and orbital motions yield dual-wavelength imaging over the entire dark polar region. New features of the global auroral morphology were discovered with this initial satellite imaging capability^2,3^.

Several auroral imagers have since been launched, expanding from nightside auroras to dayside auroras in the sunlit portion of the earth, with emissions from the Lyman–Birge–Hopfield (LBH) auroral bands. As the global auroral distribution can provide information regarding the projection of space disturbances, satellite auroral imaging has become an indispensable tool for monitoring and forecasting space weather effects. A number of satellites with auroral imaging capability have been launched to achieve this goal, from the United States, Sweden, Russia, and Japan. China has now entered the international scene of this scientific task with the newly launched Fengyun-3D satellite, which carries a wide-field auroral imager (WAI). Zhang et al.^4^ provided a detailed description of the WAI, its optical system, spatial resolution, sensitivity, calibration including flat-field correction, and data-processing procedures. Its capability in comparison with existing auroral imagers has been tabulated. In addition, a comparison of global auroral images with two adjacent images from Defense Meteorological Satellite Program (DMSP) has shown that the WAI can indeed fill the missing gaps in continuous monitoring of global auroral distributions.
